# Comparisons of Perceived Training Doses in Champion Collegiate-Level Male and Female Cross-country Runners and Coaches over the Course of a Competitive Season

**DOI:** 10.1186/s40798-017-0105-0

**Published:** 2017-10-17

**Authors:** Kyle R. Barnes

**Affiliations:** Department of Movement Science, Grand Valley State University, 1 Campus Drive, Allendale, MI 49401 USA

**Keywords:** Training load, Intensity, Session RPE, Running, Periodization

## Abstract

**Background:**

Session rating of perceived exertion (sRPE) is a practical tool for coaches to assess internal training load of their athletes. In a sport like cross-country running, that is individual in nature, but has a team training and competition component, information about the association between external and internal load is lacking. Furthermore, there is a need for studies that examine perception of training doses across multiple training cycles including the competitive season as well as between male and female athletes.

**Methods:**

Session RPE, duration, and training load (TL_RPE_ = sRPE × duration) of 25 highly trained male and female cross-country runners and their coaches were recorded for every training session (110 days) throughout a collegiate cross-country season. Intensity (sRPE), duration, and TL_RPE_ were compared between coaches and runners by gender separately. Training sessions were also analyzed by those intended by the coaches to be easy, moderate, and hard as well as by training period.

**Results:**

Data from 3024 training sessions were collected, 62% of which were considered “easy,” 18% “moderate,” and 20% “hard.” Men and women rated coach-intended easy sessions significantly harder during each month of the season (effect size (ES) > 2.9, *p* < 0.0001). Men rated moderate intensity sessions significantly higher than coaches (ES ≥ 1.0, *p* ≤ 0.002), whereas females rated hard intensity sessions significantly lower than coaches (ES > 0.5, *p* < 0.008). There was no difference between males and coach’s hard sessions (ES < 0.07, *p* > 0.05) or females and coach’s moderate sessions (ES < 0.18, *p* > 0.05). Training intensity and TL_RPE_ tended to increase throughout the season (*p* > 0.05), with a significant increase in moderate and hard intensity sessions in the last training period (*p* < 0.001).

**Conclusions:**

The results indicate the male and female cross-country runners tend to regress to moderate intensity training throughout the cross-country season. Given the success of the athletes in this study, these results show how a simple system for monitoring training such as the sRPE method may improve control of training variables and provide a useful tool for coaches to evaluate training load placed on athletes in a simple, responsive way.

## Key Points

- There is a difference between coaches and male and female cross-country runner’s intentions and perceptions of easy, moderate, and hard intensity training sessions throughout a competitive season.
- The rationale for differences in perceptions of training intensities and loads was not fully elucidated but may be related to differences in coaching supervision during easy and hard training sessions, physiological and psychosocial factors before, during, and after training sessions, communication between coaches and athletes, and/or athlete experience.
- The sRPE method for monitoring training is a practical system coaches can implement to evaluate training load placed on athletes on a day-to-day basis, in a simple, responsive way.

## Background

Previous studies have stressed the importance of periodized training load in enhancing athletic performance and the changes in performance attributable to varying periods of hard and easy training [1–4]. These practicalities are reflected in the practice of coaches who design highly detailed periodized training programs [3, 5]. Unfortunately, although periodized training programs are in their essence quantitative, there has been great difficulty in finding a way to effectively quantitate training using a single term. Distance runners have often used their training volume (total kilometers or miles run per week) and duration as an index of training with reasonable effectiveness [2, 6]. However, measurement of training programs as training volume ignores the critical importance of intensity [6]. Therefore, athletes training for endurance performance, the use of volume of training is an inadequate tool because of the overriding importance of intensity.

In response, the session rating of perceived exertion (sRPE) method of monitoring exercise intensity was developed as a modification of the category ratio RPE method [3, 7]. The modification involved asking the subject to give a global rating of their perceived exertion for the entire exercise session ~ 30 min after the conclusion of an exercise bout rather than rating the momentary level of exertion as is the usual practice with RPE [4, 7]. When this intensity rating is multiplied by the duration of the training session, a single number similar to that of a TRIMP (training impulse) score devised by Banister et al. [8] representing the magnitude of that training session or “training load” is derived. Several papers have used the sRPE method in a variety of settings and have demonstrated its value relative to quantifying exercise training intensities [1, 7, 9–11] and as the intensity component of larger schemes of evaluating training programs [1, 3, 4, 9–11]. While sRPE method may not represent a superior way of monitoring exercise intensity compared to that of heart rate monitors and global position system tracking, many teams and clubs cannot afford the equipment or time to track, monitor, and analyze such data. Therefore, sRPE represents a practical and efficient way of monitoring the internal load of many athletes that have demands beyond that of just training and competing.

Within a training program of collegiate distance runners, balancing the desired external training load by coaches with the perceived internal training load of athletes represents unique challenges in this environment. This includes trying to optimize performance by maximizing intense training sessions with recovery sessions to elicit specific training adaptations all within the confines of class schedules, practice times, and individual characteristics of each runner [12]. In addition, it is assumed that psychosocial factors, such as an exam at school, also affect the internal training load [13, 14]. Ideally, the internal load matches the external training load, assuming that the prescribed training results in optimal performance for the athlete. However, it is impossible for coaches to be fully aware of the internal load of athlete, especially in team environments. Differences between the training programs designed by coaches and executed by individual and team sport athletes are well established [9–11, 15–25]. It is suggested that this difference is even more pronounced in team sports because training load during group exercise is difficult to control [9], thus providing a plausible explanation for a lack of improvements in performance or the incidence of overtraining syndrome, injury, illness, or maladaptation to training in high-level athletes [9, 10, 13, 23].

In sports like cross-country running, that is individual in nature, but have a team training and competition component, information about the association between external and internal load is lacking. In addition, there is a need for studies that examine perception of training doses across longer time periods that include different types of training cycles including the competitive season. Finally, there is a paucity of data comparing the perceptions of female athletes to coaches in any sport or discipline as well as limited data comparing male runners to coaches. Therefore, the purpose of this study was to investigate and compare the perceptions of training doses between coaches and male and female cross-country runners independently over a full competitive cross-country season.

## Methods

### Subjects

Twenty-five highly trained cross-country runners (13 male and 12 female) were recruited from the Grand Valley State University men’s and women’s National Collegiate Athletic Association (NCAA) Division II Cross-Country teams. The characteristics of the male and female runners were (mean ± SD) age 20.2 ± 1.4 and 19.7 ± 1.6 y., training history 6.2 ± 1.3 and 6.5 ± 1.1 y., height 176.6 ± 7.8 and 168.4 ± 6.5 cm, weight 67.9 ± 7.1 and 53.9 ± 6.0 kg, and percentage body fat 6.4 ± 1.9 and 12.4 ± 3.2, respectively. Five of the men and six of the women were All-Americans (top 40 runners at NCAA Division II Cross-Country National Championship) and as a team, the Women’s team won the NCAA Division II Cross-Country National Championship and the Men’s team was the National Runner-up.

For both the men and women’s teams, two coaches were responsible for the training programs of both teams. Athletes received a training program with a combination of easy, moderate, and hard runs each week. The athletes trained together as a team 5 days each week (6 days per week during weeks that also included a competition) under the supervision of the coaches. The days in which athletes did not train together or under the supervision of the coaches were days in which easy runs were prescribed. The study was approved by Grand Valley State University’s Human Research Review Committee (Reference #: 14-206-H) and performed in accordance with the standards of ethics outlined in the Declaration of Helsinki. All participants provided informed written consent to participate.

### Experimental Testing

Before each training session throughout the entire cross-country season (110 training days), coaches rated the intended intensity of each training session (session rating of intended exertion, sRIE) on a scale from 0 (rest) to 10 (maximal exertion) (Table 1), for both teams. Because each athlete was expected to run a different total volume of training each week, coaches gave athletes a range of duration (minutes) of training each day depending on the athlete. The duration of the training session including all *running* activities from the beginning of the warm-up period to the end of the cooldown, but excluded ancillary aspects of training such as drills, resistance training, and stretching. The sRIE was blinded to athletes, but the training prescription was presented to the athlete’s in conventional terms (e.g., distance to be run and/or number and distance of intervals to be completed). Descriptive modifiers, usually presented as a particular pace, or percentage of racing pace, were often added, particularly during “hard” high-intensity training sessions. Thirty minutes after each training session, athletes evaluated their own training session by reporting their session rating of perceived exertion (sRPE) in accord with the verbal prompt, “if a friend who did not understand the specific training expressions of athletics were to ask you how hard your training session was, how would you reply” [10] (Table 1) along with the training duration of the session using an online training log. Coaches were blinded to the athlete sRPE until after the season was over as to not interfere with the athlete’s perception of training intensities. The rationale for rating the intensity (sRPE) of the training session at least 30 min after the conclusion of training is to prevent particularly hard or easy elements late in the training session from dominating the athletes’ perception of the training session [7, 11, 26]. Ratings not reported the same day were not included in the analysis. Both coaches and athletes were given verbal and written description of procedures and were supervised on a daily basis across the cross-country season.

**Table 1:** Session Rating of Perceived Exertion (sRPE) Scale

Rating	Verbal anchor^a^
0	Rest
1	Very easy
2	Easy
3	Moderate
4	Sort of hard
5	Hard
6	
7	Very hard
8	Very, very hard
9	Near maximal
10	Maximal

^a^Subjects rated the entire training session 30 min after exercise in response to the verbal prompt “if a friend who did not understand the specific training expressions of athletics were to ask you how hard your training session was, how would you reply?”

### Analysis

Following all training sessions, multiplication of the sRPE (or sRIE from coaches) by the duration yielded a dimensionless term which we will refer to as training load (TL_RPE_), and which is conceptually (although not numerically) equivalent to the training impulse (TRIMP) score derived from heart rate monitor training [7]. Means and standard deviations were calculated for intensity (sRPE or sRIE), duration, and TL_RPE_ (intensity × duration) for both coaches and athletes by gender. Training sessions were also divided into those intended by the coaches to be “easy” (RPE < 3), “moderate” (RPE = 3–5), and “hard” (RPE > 5) [9, 10]. They were then compared between the coaches’ and athletes’ perceptions of intensity, duration, and TL_RPE_ by using a two-way ANOVA. Furthermore, data for men and women divided up by month were analyzed. Bonferroni-corrected pairwise comparisons used for post hoc analysis was included on the subjects’ ratings to determine if a significant difference exists between athletes and coaches. Statistical analyses were performed using Statistical Analysis System (SAS) version 9.4 (Cary, NC). *p* values lower than 0.05 were considered statistically significant.

To determine the magnitude of effects between athletes and coaches, a spreadsheet for post-only crossovers was used [27]. The pre-test value of the dependent variable was included as a covariate to improve precision of the estimate of the effects. Effects were estimated in percent units via log transformation, and uncertainty in the estimate was expressed as 90% confidence limits. The effect size (ES), which represents the magnitude of the difference between the two conditions in terms of SD, was calculated from the log-transformed data by dividing the change in the mean by the average SD of the two conditions. Magnitudes of effects on outcomes between coaches and athletes were evaluated non-clinically: if the confidence interval overlapped thresholds for small positive and negative values, the effect was deemed unclear; all other effects were reported as the magnitude of the observed value and were evaluated probabilistically as described previously [28]. The threshold values for assessing the magnitude of small, moderate, large, very large, and extremely large effects were 0.2, 0.6, 1.2, 2.0, and 4.0 of the between-subject standard deviation [29].

## Results

Data from 3024 training sessions were collected from 25 runners (13 male and 12 female) and two coaches, of which 62% were considered “easy”, 18% “moderate”, and 20% “hard”. There was > 99% reporting compliance from all athletes. Average intensity, duration, and TL_RPE_ over the entire cross-country season by the male athletes were 3.92 ± 1.19 arbitrary units (AU), 65.7 ± 13.5 min, and 307.7 ± 113.5 AU; and female athletes were 3.67 ± 1.02 AU, 48.4 ± 9.3 min, and 210.6 ± 74.2 AU, respectively. Average intensity and TL_RPE_ as prescribed by coaches across the cross-country season was 3.09 ± 2.43 and 238.9 ± 40.5 AU for men and 3.09 ± 2.43 and 189.4 ± 29.9 AU for women, respectively.

**Fig. 1:**
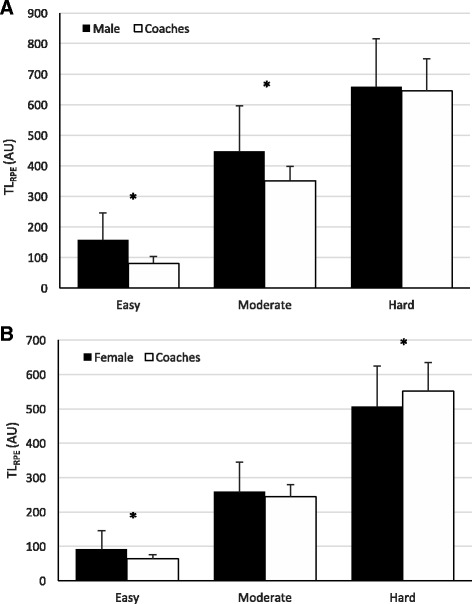
Comparison of average training load (TL_RPE_) between coaches and male runners (**a**) and between coaches and female runners (**b**) for easy, moderate, and hard training sessions throughout the cross-country season. AU arbitrary units; asterisk indicates significantly different, *p* < 0.001

Men and women rated sessions intended to be easy by the coach as very large-extremely largely higher (harder) throughout the season (men: Table 2, ES = 2.96 ± 0.46, *p* < 0.0001; women: Table 3, ES = 4.39 ± 0.48, *p* < 0.001). Males and females TL_RPE_ was also large-extremely higher (men: Fig. 1a, Table 2, ES = 1.98 ± 0.37, *p* < 0.0001; women: Fig. 1b, Table 3, ES = 3.76 ± 0.55, *p* < 0.001). Men’s intensity during moderate training sessions was largely higher (ES = 1.21 ± 0.46, *p* = 0.001) than coaches (Table 2), and TL_RPE_ was also moderately higher than coaches (Fig. 1a, Table 2, ES = 1.00 ± 0.36, *p* = 0.002) throughout the season. There was no difference between females and coach’s intensity (Table 3, ES = 0.18 ± 0.48, *p* = 0.38) or TL_RPE_ (Fig. 1b, Table 3, ES = 0.10 ± 0.46, *p* = 0.61) for moderate sessions throughout the season. There was no difference between male runners and coach’s perception of hard sessions (Table 2, ES = 0.07 ± 0.46, *p* = 0.91) whereas there was a moderate lower difference in women’s rating of hard sessions compared to coaches (ES = 0.91 ± 0.48, *p* = 0.006). There was also no difference in hard sessions TL_RPE_ for men (Fig. 1a, Table 2, ES = 0.03 ± 0.24, *p* = 0.68); however, there was a small lower difference in female’s hard session TL_RPE_ compared to coaches (Fig. 1b, Table 3, ES = 0.53 ± 0.30, *p* = 0.008).

**Table 2:** Male Runners and Coach’s Intensity (sRPE and sRIE) and Training Load (TL_RPE_) According to the Season Period and Intended Session Intensity (mean ± SD)

	Easy				Moderate				Hard			
Season period	Intensity (AU)^a^		Training load (AU)^b^		Intensity (AU)		Training load (AU)		Intensity (AU)		Training load (AU)	
	Athlete	Coach	Athlete	Coach	Athlete	Coach	Athlete	Coach	Athlete	Coach	Athlete	Coach
1	2.4 ± 0.5	1.3 ± 0.5	107 ± 74	55 ± 15	4.4 ± 0.8	3.4 ± 0.6	435 ± 141	331 ± 43	6.9 ± 0.6	6.8 ± 1.0	620 ± 167	593 ± 120
2	2.5 ± 0.5	1.1 ± 0.3	126 ± 94	48 ± 17	4.3 ± 1.2	3.4 ± 0.7	412 ± 139	321 ± 44	7.1 ± 1.0	7.1 ± 1.0	640 ± 162	630 ± 107
3	2.8 ± 0.9	1.2 ± 0.5	166 ± 86	102 ± 18	4.7 ± 0.4	3.6 ± 0.6	522 ± 170	392 ± 49	7.4 ± 0.6	7.3 ± 0.9	661 ± 149	650 ± 99
4	2.8 ± 0.9	1.3 ± 0.5	186 ± 96	98 ± 17	4.2 ± 0.2	3.8 ± 0.5	446 ± 170	386 ± 53	6.9 ± 0.6	7.7 ± 0.6	681 ± 166	705 ± 111
5^c^	2.1 ± 0.8	1.5 ± 0.6	123 ± 70	82 ± 69	5.2	5.0	572	499	10.0	10.0	768	768
Season average	2.7 ± 0.6	1.4 ± 0.5	157 ± 88	82 ± 21	4.4 ± 0.7	3.6 ± 0.6	448 ± 149	351 ± 46	7.3 ± 0.7	7.3 ± 1.0	658 ± 157	646 ± 105

*AU* arbitrary units, *sRPE* session rating of perceived exertion, *sRIE* session rating of intended exertion, *TL*_*RPE*_ training load

^a^Intensity assessed as sRPE by athletes or sRIE by coaches on 1–10 scale [10]

^b^Training load (TL_RPE_) represents dimensionless term quantified by multiplying sRPE or sRIE by duration (intensity × duration)

^c^Only one observation (training session) during season period 5 for moderate and hard training sessions

**Table 3:** Female Runners and Coach’s Intensity (sRPE and sRIE) and Training Load (TL_RPE_) According to the Season Period and Intended Session Intensity (mean ± SD)

	Easy				Moderate				Hard			
Season period	Intensity (AU)^a^		Training load (AU)^b^		Intensity (AU)		Training load (AU)		Intensity (AU)		Training load (AU)	
	Athlete	Coach	Athlete	Coach	Athlete	Coach	Athlete	Coach	Athlete	Coach	Athlete	Coach
1	2.7 ± 0.7	1.3 ± 0.5	76 ± 40	38 ± 9	3.8 ± 0.5	3.4 ± 0.6	253 ± 74	230 ± 32	6.7 ± 0.3	6.8 ± 1.0	485 ± 121	485 ± 97
2	2.3 ± 0.6	1.1 ± 0.3	72 ± 52	33 ± 10	3.7 ± 1.1	3.4 ± 0.7	244 ± 70	227 ± 26	6.6 ± 1.2	7.1 ± 1.0	491 ± 117	531 ± 84
3	2.6 ± 0.5	1.2 ± 0.5	103 ± 61	54 ± 10	3.6 ± 0.4	3.6 ± 0.6	285 ± 106	281 ± 26	7.1 ± 0.8	7.3 ± 0.9	529 ± 114	543 ± 71
4	2.5 ± 0.8	1.3 ± 0.5	105 ± 54	63 ± 14	3.5 ± 0.3	3.8 ± 0.5	261 ± 113	286 ± 51	7.2 ± 1.0	7.7 ± 0.6	511 ± 125	548 ± 84
5^c^	2.0 ± 0.7	1.5 ± 0.6	77 ± 57	56 ± 15	5.2	5.0	328	335	9.4	10.0	585	627
Season average	2.5 ± 0.7	1.3 ± 0.5	92 ± 53	64 ± 12	3.7 ± 0.6	3.6 ± 0.6	259 ± 87	244 ± 34	6.9 ± 0.9	7.3 ± 1.0	507 ± 118	551 ± 84

*AU* arbitrary units, *sRPE* session rating of perceived exertion, *sRIE* sessions rating of intended exertion, *TL*_*RPE*_ training load

^a^Intensity assessed as sRPE by athletes or sRIE by coaches on 1–10 scale [10]

^b^Training load (TL_RPE_) represents dimensionless term quantified by multiplying sRPE or sRIE by duration (intensity × duration)

^c^Only one observation (training session) during season period 5 for moderate and hard training sessions

**Fig. 2:**
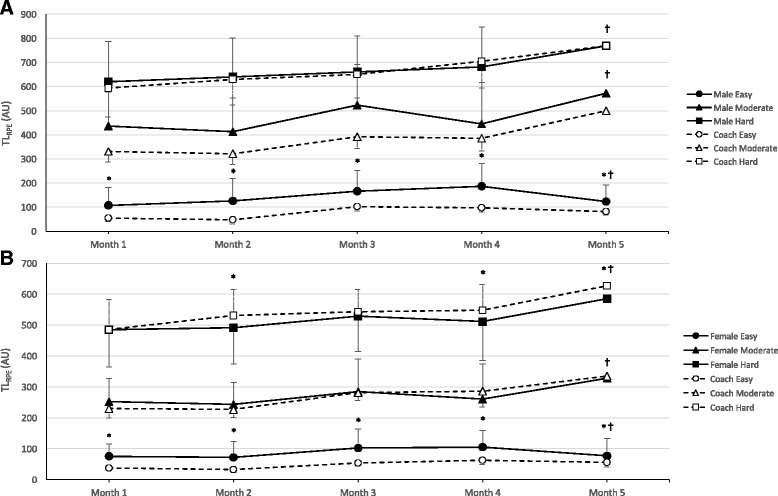
Comparison of training load (TL_RPE_) between coaches and male runners (**a**) and between coaches and female runners (**b**) for easy, moderate, and hard sessions during each month of the cross-country season (1–5). Month 5 only included one moderate session and one hard session. AU arbitrary units; asterisk indicates significantly different between athlete and coach within respective month, *p* < 0.01; dagger indicates significantly different than month 4, *p* < 0.01

When examining the breakdown of males and female’s intensity (Tables 2 and 3) compared to the coaches by month over the duration of cross-country season, results revealed significant differences between both males and female sRPE and coach sRIE during each month of the season for easy sessions (*p* < 0.0001). There were also significant differences in easy training session TL_RPE_ between gender and coach during each month of the season (Fig. 2, Tables 2 and 3, *p* < 0.001). The TL_RPE_ for easy sessions tended to increase from month 1 to month 4 throughout the season (*p* > 0.12) but significantly reduced from month 4 to 5 (*p* = 0.005) for both males and females (Fig. 2). There were no significant differences between male athletes and coaches for moderate or hard sessions each month in regard to rating of intensity (Table 2, *p* > 0.05) or TL_RPE_ (Fig. 2, Table 2, *p* > 0.05). However, months 1–3 for men’s intensity approached statistical significance (*p* = 0.17, *p* = 0.06, *p* = 0.31, respectively). There were also no differences between females and coach’s moderate intensity or TL_RPE_ (*p* > 0.05) during any month. However, during months 2, 4, and 5, there were significant differences in intensity and TL_RPE_ during hard sessions (*p* < 0.01) between female athletes and coaches. Session RPE and TL_RPE_ for moderate sessions tended to undulate from month to month throughout the season; however, there was a significant (*p* < 0.001) increase in moderate intensity TL_RPE_ during month 5 for both males and females (Fig. 2). Similarly, hard sessions tended to increase (*p* > 0.05) during each month throughout the season with a significant (*p* < 0.001) increase in month 5 (the NCAA Cross-Country National Championship Competition) respective to month 4 (Fig. 2).

## Discussion

The aim of the current study was to investigate and compare the perceptions of training dose between coaches and male and female cross-country runners over a full competitive cross-country season. In general, athletes tended to perceive training as closer to moderate intensity throughout the season regardless of the intended session intensity by the coach. Likewise, for coach-intended easy and moderate sessions, male and female athletes reported higher intensity and training loads. For intended hard days, there was no difference between male runners and coaches, whereas females were significantly lower.

The discrepancy between runners’ and coaches’ perceptions of TL_RPE_ in the current study depended on the training intensity. Previous studies have shown during hard training sessions (i.e., RPE > 5), the sRPE and TL_RPE_ experienced by the athletes are lower than planned by the coaches [9, 10, 15, 18, 19, 23, 25]. Our results agree with this finding. Female runners consistently reported lower sRPE and TL_RPE_ than coaches during hard sessions throughout the season (Fig. 2, Table 3), while male runners varied from month to month (Fig. 2, Table 2) but on average there was no difference (ES = 0.03, *p* = 0.68) throughout the season (Fig. 1). The discrepancy between athletes and coaches could be psychophysiological in nature. The performance of athletes is enhanced primarily as a response to high-intensity and/or long duration training sessions [10]. Therefore, often times in running, the athletes are informed ahead of time by the coaches what the hard workouts are going to be to prepare mentally and physically for such sessions. In the build-up to these hard sessions, athletes may actually mentally prepare for hard workouts to be harder than they actually are and, upon completion, realize it was easier than anticipated. Alternatively, [9] suggested that coaches may actually overprescribe the intensity of hard training sessions because the overall load of the training program may be too high.

Another explanation for the small to negligible differences during moderate to hard session in our study could be the fact that of the 25 runners in the study, all but two had been under the guidance of the same coaches for at least 2 years and all athletes had been performing run-specific training for at least 5 years. Previous research has shown greater agreement in perception of training intensity in experienced versus inexperienced athletes [9, 15]. It would appear that for these athletes, training in the same system and under the supervision of the same coaches for 2 to 4 years has taught them how training is communicated on a daily basis and they trust the coach’s expectations in terms of duration and intensity of each hard training session. Stewart and Hopkins [23] showed that trained swimmers also complied with prescribed distances and rest intervals during hard training sessions but were less effective in judging the intensity of swim training. This is not the case in all training programs, such that runners in another study regularly failed to comply with coach expectations in regard to the distance to be run and pace desired [10].

Accordingly, during easy (i.e., RPE < 3) and moderate (i.e., RPE 3–5) sessions, sRPE and TL_RPE_ were greater than that intended by the coaches. This tendency toward perception of moderate training loads is similar to that reported previously in other endurance runners and swimmers [10, 15, 25] as well as soccer players [9]. Possibly, this discrepancy between athletes and coach’s perceptions of intensity and TL_RPE_ during easy and moderate sessions could be related to differences in supervision of athletes during these training sessions compared to hard sessions. In this study, the coaches were physically present during the entire hard training sessions whereas during easy and moderate sessions athletes were often given instructions and sent out for the majority of their run without supervision. It could be that the athletes in this study tended to overtrain on easy and moderate days when the coaches were not present to adjust their intensity or groups each athlete was running with accordingly. The fact that cross-country is a team sport and only a finite number of runners can compete in championship events may also partially explain this phenomenon. To convince the coach, runners may feel the need to perform above desired training intensities to show their superior fitness levels compared to teammates [9]. Since easy or moderate training sessions typically follow hard sessions, another explanation may be that coaches may have a misconception of the athlete’s physiological state following the previous session load(s) [19]. Although coaches expect an easy training session the day following a hard session, it is possible that athletes are not recovered physically or psychologically enough to perceive such training as easy.

In collegiate cross-country running, while the training and racing are performed as an individual athlete, workouts regardless of intensity are often executed in small groups, making it easier for coaches to plan, control, and adjust exercise intensity depending on the day. With the planning of training and attempting to optimize performance on race day, coaches should keep in mind the physical and psychosocial characteristics that affect the internal load of each runner on the team. How to target intended training load is a complex procedure for coaches and requires an accurate analysis of data collection through objective and subjective observations of the athlete both in real-time and within the context of the present, past, and future training blocks, while also taking into account their own experience and training philosophy [12, 20]. Accordingly, runners in the present study were regularly relegated to other training groups throughout the season and within training sessions themselves based on observations from when the coaches were present, particularly during moderate and hard sessions.

The tendency for athletes to regress toward perceptions of moderate training loads may from a theoretical standpoint have important implications for training athletes [9, 10, 25]. It has been previously suggested that this decrease in the day-to-day variability in training load may increase the risk of overtraining, injuries, illness, and maladaptation to the previous training sessions [10, 23]. Although injuries, illness, or markers of overtraining syndrome were not recorded in the present study, there were several incidents throughout the season that caused athletes to miss multiple training days. Whether these occurrences were correlated to or caused by the regression to moderate training loads is unknown; however, we can note that none of the athlete’s experiences forced them to miss the remainder of the season. Furthermore, we found no reason to believe the athletes experienced any sort of decrements in running performance, particularly during the latter parts of the season given the success of the two teams discussed earlier.

## Conclusions

In conclusion, our results indicate that there is a systematic difference between coaches and male and female cross-country runner intentions and perceptions of easy, moderate, and hard intensity training sessions throughout a competitive season. The rationale for this has not been fully elucidated but may be related to differences in coaching supervision during easy and hard training sessions, physiological and psychosocial factors before, during, and after training sessions, communication between coaches and athletes, and athlete experience. Given the success of the athletes in this study, these results show how a simple system for monitoring training such as the sRPE method may improve control of training variables and provide a useful tool for coaches to evaluate training load placed on athletes on a day-to-day basis, in a simple, responsive way [4, 25]. Future studies should examine the link between perceptions of effort following moderate to hard training sessions or following long durations of training sessions.

## Abbreviations

ANOVA: Analysis of variance
AU: Arbitrary units
ES: Effect size
NCAA: National Collegiate Athletics Association
RPE: Rating of perceived exertion
SD: Standard deviation
sRIE: Session rating of intended exertion
sRPE: Session rating of perceived exertion
TL_RPE_: Training load
TRIMP: Training impulse
